# Pacific Railroad

**Published:** 1866-09

**Authors:** 


### PACIFIC RAILROAD.

SUCH is the conformation of the earth's surface and the relative position of the countries of the world in reference to trade, commerce, and manufacture, that the great pathway of nations is destined to be that which connects the Mississippi river with the western shore of the Pacific Ocean, by rail.

As between China, Japan, Australia, and the East Indies, on the one hand, and the United States, with England and Western Europe, on the other, the Pacific railway would save months of time in transit—and time is money—hence the road will be built. Not one line, but two; for two are imperatively required. The necessities of the times, martial, civil, social, and commercial, will have them, and that too, in a much briefer space than most persons imagine. One from Oregon to St. Anthony's Falls; the other from San Francisco by way of Texas to New Orleans. Then, by reason of the trade winds going and returning across the Pacific, the quickest route for travel or freight, from the Americas, will be from New Orleans via El Passo, in Texas, to San Francisco; while from China and adjacent countries, to the United States and England, the most expeditious conveyance will be across the Pacific, and by way of the line from the mouth of the Columbia or Puget Sound to St. Anthony's Falls, Minnesota, or the Mississippi river. A round voyage thus, will save between one and two months' time; and that, on a cargo worth millions of money, besides wages, is an item so considerable, that private enterprise will greedily seize at the chance of investment.

